# Effects of Electroacupuncture on Opioid-Induced Constipation in Patients With Cancer: Study Protocol for a Multicenter Randomized Controlled Trial

**DOI:** 10.3389/fmed.2022.818258

**Published:** 2022-04-13

**Authors:** Weiming Wang, Xinlu Wang, Yan Liu, Yuanjie Sun, Xiaoxu Liu, Yan Yan, Zhishun Liu

**Affiliations:** ^1^Department of Acupuncture and Moxibustion, Guang'anmen Hospital, China Academy of Chinese Medical Sciences, Beijing, China; ^2^Key Laboratory of Chinese Internal Medicine of Ministry of Education, Dongzhimen Hospital, Beijing University of Chinese Medicine, Beijing, China

**Keywords:** opioid-induced constipation, acupuncture, cancer patients, randomized controlled trial, spontaneous bowel movements

## Abstract

**Background:**

Opioid-induced constipation (OIC) is one of the most prevalent adverse events associated with cancer patients who receive opioid analgesics for moderate to severe pain. Acupuncture may be an effective treatment for OIC. We designed this trial to assess the efficacy and safety of electroacupuncture for OIC in cancer patients.

**Methods:**

This is a multicenter, sham-controlled, parallel-group, subject- and assessor-blinded randomized trial. A total of 100 cancer patients with OIC will be randomly assigned to either the electroacupuncture group or the sham electroacupuncture group at a ratio of 1:1. Patients in each group will receive a total of 24-session treatment over 8 weeks, three sessions a week and 30 min each session. Thereafter, patients will be followed up for another 8 weeks. The primary outcome will be the proportion of responders, defined as a patient who has ≥3 spontaneous bowel movements (SBMs)/wk and ≥ increase of 1 SBM from baseline simultaneously for at least 6 out of 8 weeks of the treatment period. The secondary outcomes will include the mean weekly SBMs and complete spontaneous bowel movements, the mean Bristol Stool Form Scale score for stool consistency, the mean score for straining of SBM, the total and subscale scores of Patient Assessment of Constipation-Symptom questionnaires, and the total and subscale scores of Patient Assessment of Constipation-Quality of Life questionnaire. Patients' global assessment of treatment effectiveness, patients' expectation toward the effectiveness of acupuncture and safety of acupuncture will also be assessed. All efficacy analyses will be performed in the intention-to-treat population.

**Discussion:**

To improve the adherence to intervention protocols, the majority of the participants will be recruited from an inpatient setting. The results will help to determine the clinical effects and safety of electroacupuncture for the treatment of OIC among patients with cancer.

**Clinical Trial Registration:**

www.ClinicalTrials.gov, identifier: NCT03797586, registered on 4 January 2019, https://clinicaltrials.gov/ct2/show/NCT03797586.

## Background

Approximately 70–80% of patients with advanced diseases are tortured by moderate to severe pain (1). Opioid analgesics, such as morphine and oxycodone, are recommended as the cornerstone for the management of moderate to severe cancer pain by WHO Cancer Pain Relief Guidelines (2, 3). Systemic opioid therapy is recommended by some studies to cancer patients with moderate to severe pain, regardless of the underlying mechanisms of the pain (4). Opioid analgesics can activate receptors in both the central nervous system (CNS) and the peripheral nervous system, thus relieving pain and improve patients' quality of life (5). However, these drugs can lead to serious adverse events (AEs), with an overall rate ranging from 1.8 to 13.6% (6, 7), of which opioid-induced constipation (OIC) is the most prevalent one. OIC is defined as a change in baseline bowel habits or defecatory patterns following the initial administration or modification of opioid therapy (8–10). It is reported in 41% of non-cancer patients (11) and 94% of cancer patients who take opioids for pain (12). Unlike many other opioid-related AEs, the symptoms of OIC tend to be persistent and difficult to tolerate (9), which can adversely reduce patients' quality of life (8, 13–15) and result in dose reduction or discontinue of opioid analgesics eventually (16). The mechanism of OIC involves multiple contributing factors (17): exogenous opioids can activate μ-receptors throughout the gastrointestinal tract and lead to a change in gut motility, a decrease in gut secretion and an increase in sphincter tone, which will result in OIC (18). The management of OIC is multifaceted (19), involving a combination of pharmacological and non-pharmacological interventions, such as laxatives and increased fluid and fiber intake (8, 9). However, the efficacy of these interventions is limited and these approaches do not address all of the underlying pathophysiological mechanisms of OIC (8, 9). Recently, peripherally acting μ-opioid receptor antagonists (PAMORAs), such as naloxegol and methylnaltrexone, have been shown to be effective in treating OIC patients who response poorly to simple laxatives (20). However, these drugs are still under test in clinical trials with unclear long-term efficacy and safety; they have not been approved for use in China. In addition, the use of PAMORAs is often accompanied by AEs of abdominal pain and flatulence (21). Therefore, it is still necessary to explore new approaches for the treatment of OIC.

Acupuncture, a traditional Chinese medicine approach, has been used to treat gastrointestinal disease, including constipation, for thousands of years. Two systematic reviews concluded that acupuncture can improve spontaneous bowel movement (SBMs) in functional constipation (22, 23). Furthermore, our recent study indicated that electroacupuncture (EA) could increase complete spontaneous bowel movements (CSBMs) and SBMs, revealing a long-term effect of 24 weeks after treatment ceased and has good safety profile among patients with chronic severe functional constipation (24, 25). Acupuncture can facilitate the gastrointestinal motility and improve gastrointestinal function *via* activating somatic and peripheral nerves, sending an afferent signal to the nucleus tractus solitarii, and then resulting in an enhanced vagal efferent flow to the GI tract (26). Currently, there is still a lack of evidence regarding the efficacy of acupuncture for OIC. The objective of this study is to assess the efficacy and safety of EA compared to sham acupuncture (SA) in the treatment of OIC in patients with cancer.

## Methods

### Study Design

**Figure 1:**
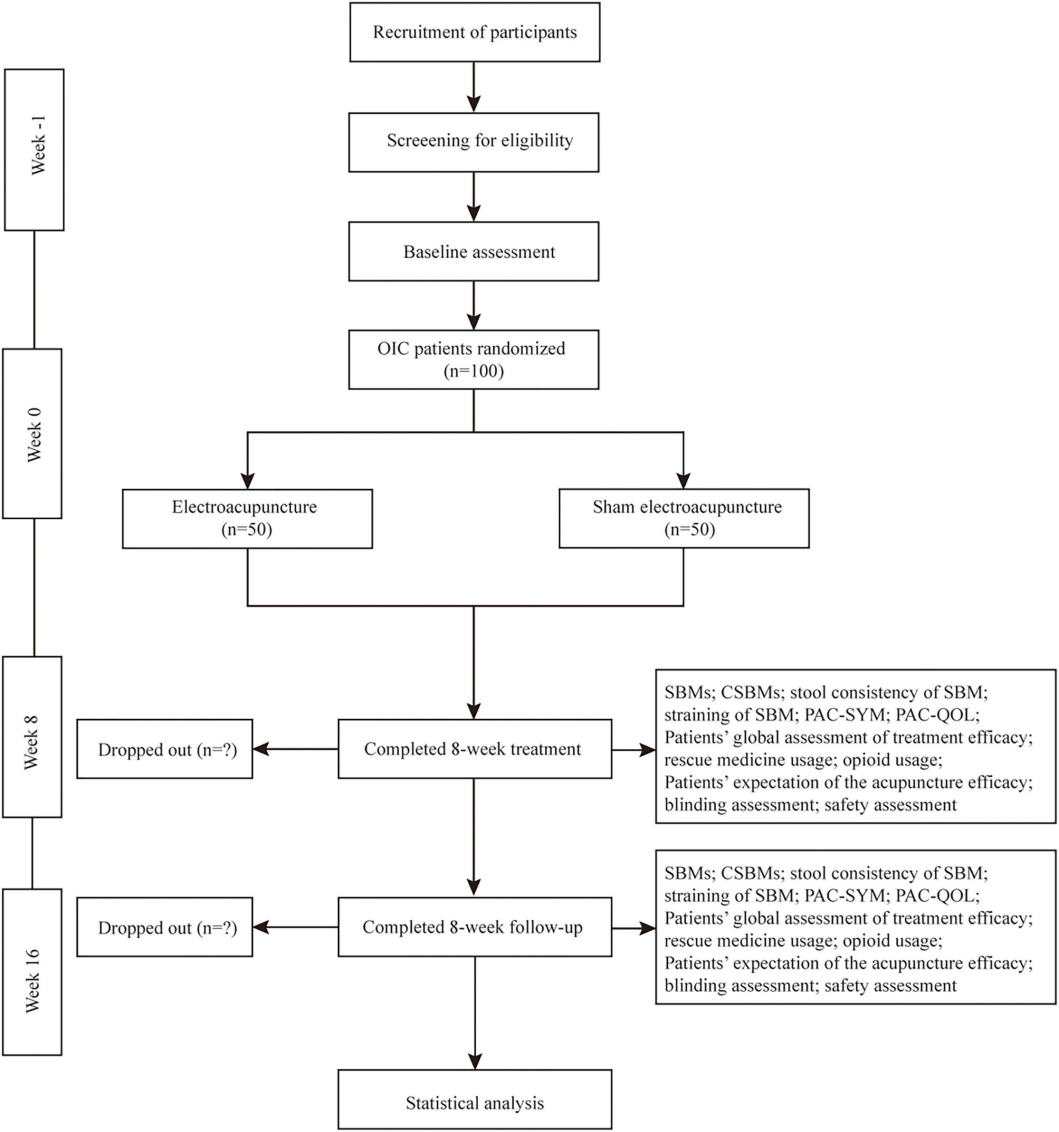
Trial flow diagram. OIC, Opioid-induced constipation; SBMs, spontaneous bowel movements; CSBMs, complete spontaneous bowel movements; PAC-SYM, Patient Assessment of Constipation-Symptom questionnaires; PAC-QOL, Patient Assessment of Constipation-Quality of Life questionnaires.

This is a multicenter, prospective, sham-controlled, parallel-group, subject- and assessor-blinded, randomized, superiority trial. The protocol of this study was developed according to the Recommendations for Interventional Trials (SPIRIT) (27) and the Standards for Reporting Interventions in Clinical Trials of Acupuncture (STRICTA) (28) guidelines. The flow chart and the time frame of assessment are shown in the Figure 1 and Table 1, respectively.

**Table 1:** The time frame of assessment.

	**Study period**			
	**Baseline**	**Allocation**	**Treatment**	**Follow-up**
	**Weeks−1**	**Week 0**	**Weeks 1–8**	**Weeks 13–16**
**Enrollment**				
Eligibility criteria	×			
Demography characteristics	×			
Disease history of cancer	×			
Disease history of OIC and constipation	×			
Eligibility screen	×			
Informed consent	×			
Allocation		×		
**Interventions**				
Electroacupuncture			×	
Sham electroacupuncture			×	
**Assessments**				
SBMs	×		×	×
CSBMs	×		×	×
Mean Bristol Stool Form Scale score for stool consistency of SBM	×		×	×
Mean score for straining of SBM	×		×	×
PAC-SYM total score and subscale scores	×		×	×
PAC-QOL total score and subscale scores	×		×	×
Patients' global assessment of treatment efficacy			×	×
Rescue medicine usage	×		×	× (weeks 9–16)
Opioid usage	×		×	×
Patients' expectation of the acupuncture efficacy	×			
Blinding assessment			×	
Adverse events	×		×	×
Safety assessment	×		×	×

*OIC, Opioid-induced constipation; SBMs, spontaneous bowel movements; CSBMs, complete spontaneous bowel movements; PAC-SYM, Patient Assessment of Constipation-Symptom questionnaires; PAC-QOL, Patient Assessment of Constipation-Quality of Life questionnaires*.

### Study Setting and Recruitment

This trial is scheduled to be performed at six hospitals in China mainland from May 2019 to October 2022. A total of 100 OIC participants with cancer will be publicly recruited from inpatient and outpatient departments through posters and networks in the Guizhou University of Traditional Chinese Medicine, Zhejiang Hospital, Jiangsu Provincial Hospital of Traditional Chinese Medicine, Hengyang Hospital Affiliated to Hunan University of Chinese Medicine, Wangjing Hospital affiliated to China Academy of Chinese Medical Sciences, and Yantai Hospital of Traditional Chinese Medicine. The study will use competitive recruitment. Guang'anmen Hospital affiliated to China Academy of Chinese Medical Sciences will be responsible for study design, data interpretation and writing of report. The duration of the trial for each participant will be 17 weeks: 1- week baseline assessment (run-out period), 8- week treatment, and 8- week follow-up.

### Randomization and Blinding

Web-based central randomization will be performed by the Linkermed Pharm Technology Co. Ltd (Beijing, China). Participants will be randomly allocated, in a 1:1 ratio, to either the EA or the SA group using permuted block-randomization and stratified by center. Acupuncturists at each site will log in the central randomization system to apply for the randomization number and group allocation. Participants, outcome evaluators, and data analysts will be blinded to the group assignments. The acupuncturists who perform the treatment will not be blinded due to the nature of the acupuncture treatment.

### Patients

One hundred cancer patients with OIC will be included in the study. Research assistants will be in charge of recruitment. Researchers will inform the participants of the possible benefits and risks associated with this trial. Oncologists or gastroenterologists will be responsible for diagnosing OIC and evaluating the status of participant (e.g., life expectancy). Participants will be required to sign informed consent before enrolment and will be free to withdraw from the trial at any time.

### Inclusion Criteria

(1) Cancer patients must meet the Rome IV (10) diagnostic criteria for OIC. Participants have at least 2 of the following new or worsening symptoms of constipation following initiation, alteration, or increase in opioid treatment: fewer than three SBMs per week, straining (>25% of defecations), sensation of incomplete evacuation (>25% of defecations), lumpy or hard stools (>25% of defecations), and/or sensation of anorectal obstruction/blockage (>25% of defecations). For patients with a history of chronic functional constipation, he/she must have worsening symptoms of constipation when the opioid therapy is initiated, changed, or the dose is increased;
(2) Patients recruited in this trial must have a history of OIC symptoms for at least 1 week;
(3) Patients must be ≥18 years of age and ≤ 85 years of age;
(4) Patient's cancer condition must be stable with a life expectancy that is more than 6 months;
(5) Patients must have an Eastern Cooperative Oncology Group (ECOG) (29) performance status of 0–3;
(6) Patients must have been receiving a stably maintained opioid regimen, consisting of a total daily dose of 30–1,000 mg oral morphine equivalents for at least 2 weeks prior to screening for cancer pain. Furthermore, it must be anticipated that the opioid will be maintained for at least 10 weeks;
(7) The SBM frequency of the patients must be ≤ 2 times a week when laxatives are not being taken;
(8) Patients must be capable of oral intake of drugs, food and beverages;
(9) Provision of written informed consent before inclusion.

### Exclusion Criteria

Patients who fulfill any of the following criteria will be excluded:

(1) Patients diagnosed with clinically significant abnormal defecation due to functional disorders or structural abnormalities of the gastrointestinal tract and other tissues related to gastrointestinal tract (not including OIC): inflammatory bowel disease, rectal prolapse, gastrointestinal obstruction, peritoneal metastasis, or peritoneal tumor at the time of enrollment;
(2) Patients with a history of gastrointestinal tract operation, abdominal operation, or abdominal adhesion within 1 month prior to screening; history of intestinal obstruction within 3 months prior to screening;
(3) Diagnosis of active diverticular disease; or severe hemorrhoid; or anal fissure; or artificial rectum or anus;
(4) Patients with an intraperitoneal catheter or those that use a feeding tube to maintain vital signs;
(5) Diagnosis of pelvic disorder, which are considered to have obvious effects on the intestinal transport of feces [such as uterine prolapse ≥degree 2, uterine fibroids (located in the posterior of the uterus with a diameter ≥ 5 cm) affecting bowel movement];
(6) Patients that are being treated with a new cancer chemotherapy, which had never been administered in the past, within 14 days of the screening or are scheduled to receive such therapy during the study;
(7) Patients that received radiotherapy within 28 days of the screening or are scheduled to receive such therapy during the study;
(8) Patients that underwent a surgery or intervention that is considered to have an obvious effect on the gastrointestinal functions within 28 days of the screening or are scheduled to receive surgery or intervention which is considered to have obvious effects on the gastrointestinal functions during the study, or scheduled to receive surgery or intervention which will be anticipated to prevent the patients from completing the trial;
(9) Patients with uncontrolled hyperthyroidism, severe hypertension, heart disease, systematic infection or blood coagulation disorders (hypercoagulation status or hemorrhagic tendency);
(10) Patients that consumed >4 additional opioid doses per day, for breakthrough pain, for more than 3 days during the baseline period, or if their maintenance opioid dosing regimen was modified during this period;
(11) Patients with severe cancerous pain [e.g., typical average daily pain intensity rating of 7–10 on a numerical rating scales (NRS; 0 (no pain) to 10 (the worst pain possible)) after the utility of routine dose and frequency of opioids] refractory to opioid therapy;
(12) Patients with a history of opioid discontinuation due to severe adverse events or patients that are suspected to discontinue opioid use due to the potential risk of adverse events;
(13) Patients that received an opioid receptor antagonist or agonist within 1 month of the screening, or those who are scheduled to receive such therapy during the study;
(14) Patients with a history of nerve neurolysis;
(15) Patients with severe cognitive impairment, aphasia, or psychiatric disorders; abdominal aortic aneurysm; hepatomegaly; or splenomegaly;
(16) Patients that have received acupuncture within 3 months of the screening;
(17) Other patients who are considered ineligible for the study by the investigator on the basis of concomitant therapy and medical findings.

## Intervention and Comparison

### EA Group

Acupuncturists who had an acupuncture license and at least 2 years of clinical experience in acupuncture will perform the treatment. We will use disposable acupuncture needles (of the following sizes: 0.30 × 40, 0.30 × 50, and 0.30 × 75 mm) and SDZ-V EA apparatus (all Hwato Brand, Suzhou Medical Appliance Factory, Suzhou, China) in this trial. The planned treatment protocol is based on our previous trials regarding acupuncture for functional constipation (24, 30). Bilateral Tianshu (ST25), Fujie (SP14), Shangjuxu (ST37) will be used in the EA group. The location of the acupoints will be based on *Nomenclature and location of acupuncture points* (31) drafted in 2006 by the National Standard of the People's Republic of China (GB/T 12346–2006).

The local skin will be routinely sterilized while the patient is in a supine position. For ST25 and SP14, 0.30 × 50 mm or 0.30 × 75 mm needles will be gently vertically inserted to the muscle layer of the abdominal wall, where patients will feel sharp pain and acupuncturists will feel resistance from the needle tip. For ST37, 0.30 × 40 mm needles will be vertically inserted ~15 mm deep, followed by three-time manipulation of even lifting and twisting method to elicit the sensation of deqi (32). Paired alligator clips of the EA apparatus will then be attached to the needle holders of the bilateral ST25, SP14, and ST37. The stimulation will be retained for 30 min, with a continuous wave of 10 Hz and current intensity of 0.5 to 4 mA. All needles will be removed after 30 min and pressure will be applied using a dry sterilized cotton ball to avoid bleeding. Patients will be followed up for another 8 weeks after the treatment stopped.

### SA Group

The patients in the SA group will receive minimal needling at non-acupoints as bilateral sham ST25, SP14, and ST37. The sham ST25 and SP14 are located 2 cm horizontally outward of the points stimulaed in the EA group. The sham ST37 point is located outward of ST37 in the middle of the stomach and gallbladder channel. After sterilization of the skin, 0.30 × 40 mm needles will be directly inserted about 2–3 mm until they can stand up when attached by the alligator clips. No manipulation will be used and no deqi sensation will be elicited at any of the sham points. The bilateral sham ST25, SP14, and ST37 points will be attached by the same EA apparatus with a continuous wave of 10 Hz and current intensity of 0.1–0.2 mA for 30 min with only the initial 30 s on.

Patients in both groups will receive 24 treatment sessions over an 8-week period (three sessions each week, ideally every other day). Each session will last for 30 min. Patients will be treated separately to prevent between-patient communication. Patients will be followed up for another 8 weeks after the treatment stopped.

### Rescue Medication

During the trial, other medication or intervention for OIC will be discouraged. However, if a patient has no bowel movement for 72 consecutive hours, only bisacodyl (5–10 mg; up to 20 mg per day) or a 110 ml glycerol enema will be permitted as a rescue medication. Details of drug use (time and frequency) will be recorded.

### Outcome Measures

The patients will be asked to keep a patient diary every day for 13 weeks: 1 week as baseline before randomization (run-out period before baseline), 8 weeks during the treatment period and 4 weeks (weeks 13–16) during the follow-up period. The content of the diary includes the bowel movements, the stool consistency, degree of difficulty in defecation, the rescue medicine drugs and duration of usage, and the intensity of cancerous pain. The diary will be collected weekly during the 8-week treatment period and collected at the end of week 16 during the follow-up period. The outcome evaluators will examine the contents of the diary and determine the SBM and the frequency accordingly.

### Primary Outcome

The primary outcome of this study is the proportion of responders, defined as a patient that has ≥3 SBMs/wk and ≥ increase of 1 SBM from baseline simultaneously for at least 6 out of 8 weeks of the treatment period. SBM is defined as a bowel movement that occurred without any medication or intervention to assist within the previous 24 h (33). A bowel movement that occurs within 24 h of an optional assisted method (rescue medication or other bowel-treatment regimens) for defecation is not considered to be an SBM.

### Secondary Outcomes

The secondary outcomes of this study include the following items:

(1) Changes in the mean weekly SBMs from the baseline during weeks 1–8 and 13–16. The mean weekly SBMs equals the total frequency of SBMs divided by the numbers of week(s) recorded;
(2) The proportion of patients with ≥3 mean weekly SBMs during weeks 1–8 and 13–16;
(3) The proportion of patients with an increase of ≥1 mean weekly SBM from the baseline during weeks 1–8 and 13–16;
(4) A change in the mean weekly CSBMs from the baseline during weeks 1–8 and 13–16. A CSBM is defined as an SBM with the feeling of complete evacuation (33). The mean weekly CSBMs equals the total frequency of CSBMs divided by number of week(s) recorded;
(5) The proportion of patients with ≥3 mean weekly CSBMs during weeks 1–8 and 13–16;
(6) The proportion of patients with an increase of ≥1 mean weekly CSBM from the baseline during weeks 1–8 and 13–16;
(7) A change in the mean Bristol Stool Form Scale score for stool consistency of SBMs from the baseline during weeks 1–8 and 13–16. For stool consistency, each patient will be asked to record their stool consistency according to the Bristol Stool Form Scale (34) on the following seven points scale (scored from 1 to 7 for stool types 1–7, respectively);
(8) A change in the mean score for the straining of SBMs from the baseline during weeks 1–8 and 13–16. For assessment of the straining of SBMs, each patient will be asked to rate his/her score of straining, using the following five-point scale (35): not at all difficult (0), a little bit difficult (1), moderately difficult (2), quite a bit difficult (3), extremely difficult (4);
(9) A change in the total and subscale score of the Patient Assessment of Constipation-Symptom (PAC-SYM) questionnaire from the baseline at weeks 8 and 16. The PAC-SYM is a questionnaire used to evaluate the severity of chronic constipation in the past 2 weeks. It consists of 12 items, which are subdivided into abdominal (four items), rectal (three items), and stool (five items) scales (36). The score of each item ranges from 0 to 4, with 0 = symptom absent, 1 = mild, 2 = moderate, 3 = severe, and 4 = very severe. Lower scores indicate a lower symptom burden. Each subscale score will be calculated as the mean of the completed items for that subscale. The total score will be calculated as the mean of all completed items. In this trial, the Chinese version of PAC-SYM, which has been validated to have a satisfactory psychometric property (37), will be used;
(10) A change in the total and subscale scores of the Patient Assessment of Constipation-Quality of Life (PAC-QOL) questionnaires from the baseline at weeks 8 and 16. The PAC-QOL is a 28-item self-reported questionnaire to assess the burden of constipation on patients' everyday functioning and wellbeing in the 2 weeks (14 days) prior to assessment (38). This questionnaire is divided into four subscales: physical discomfort (items 1–4), psychosocial discomfort (items 5–12), worries/concerns (items 13–23), and satisfaction (items 24–28). Each of the item scores ranges from 0 (not at all) to 4 (extreme), with lower scores indicating a better quality of life. For each visit, individual subscale scores will be calculated as the mean of the completed items for that subscale. The total score will be calculated as the mean of all of the completed items. We will use the Chinese version of this test (39) in our trial, which has been demonstrated to be a reliable and valid tool;
(11) Patients' global assessment of treatment efficacy. Each patient will be asked to rate his/her efficacy of treatment using the following 7-point self-reporting scale: markedly worse (1), moderately worse (2), slightly worse (3), no change (4), slightly improved (5), moderately improved (6), and markedly improved (7). Scales with seven response categories are easy to use and have shown a high reliability and validity (40). This questionnaire will be completed at week 8 and 16;
(12) The proportion of patients using rescue medicine and the mean frequency of rescue medicine use per week during weeks 1–8 and 9–16; Other Pre-specified Outcome Measures
(13) The proportion of patients discontinuing the opioid, and those with a ≥30% weekly mean increase or decrease in the dose of opioid from baseline during weeks 1–8 and 9–16.
(14) The proportion of patients with a change from baseline in anti-tumor therapy that could impair the defecation during weeks 1–8 and 9–16.
(15) Patients' belief in the efficacy of acupuncture. Participants will be asked to answer the following questions at baseline: “Do you think acupuncture will be effective in treating the disease in general?” and “Do you think acupuncture will be effective in improving the OIC?” For each question, patients will choose one of the following answers: “unclear/whatever,” “Yes,” or “No;”
(16) Blinding assessment. The blinding is regarded as successful when a patient guesses EA is the acupuncture modality they have received. Patients will be informed before the randomization that they will have a chance of 50% to receive EA or SA. They will also be told that the electrical stimulation in both groups are relatively weak, and they may fail to sense the stimulation gradually during the treatment process out of the tolerance in human body. Within 5 min after any treatment in the 8th week, the patients will be asked to answer the following question: “Is EA the acupuncture modality that you have received?” Patients will choose one of the following answers: “Yes,” or “No.”

### Safety Assessment

All AEs will be recorded throughout the whole trial in Adverse Event Form (AEF) by patients themselves and outcome assessors. AEs will be categorized as treatment-related (e.g., dizziness, fainting, localized hematoma, localized minor infection, or some discomforts after acupuncture) or non-treatment-related. Detailed information regarding AEs and serious adverse events (SAEs)—including the name, onset date, intensity, relationship with acupuncture and outcome—will be recorded. Safety assessments also include an 11-point NRS to evaluate the intensity of cancer pain. The mean and largest intensity of cancer pain during the preceding week will be evaluated at baseline, as well as weeks 2, 4, 6, 8, and 16. SAEs (e.g., any event resulting in death, requiring hospitalization, causing disability or impaired ability to work), if occurs, will be immediately reported to the principle investigator and the Medical Ethics Committee of Guang'anmen Hospital, based on which a decision to terminate or adjust the trial will be made.

### Sample Size Calculation

Assuming a responder proportion (based on the unpublished preliminary data collected from 10 participants with EA group and 10 SA controls) of 45.4% in the EA group and 14.0% in the SA group, we estimated that a sample size of 100 patients will provide 90% power to detect a 31.4% or greater difference in responder proportions between groups with at a two-sided alpha level of 0.05 and 20% loss to follow-up.

### Statistical Analysis

All efficacy analyses will be performed in the intention-to-treat (ITT) population, which is defined as all randomized participants. Safety analyses will be conducted among randomized patients who received at least one treatment session. Missing data on the primary outcome will be imputed using the multiple imputation method under the missing at random assumption. In case of relevant differences in baseline variables between the two groups, those unbalanced variables will be used as covariates for the analysis of the primary outcome. Subgroup analyses of efficacy will be performed according to individual opioid dose at baseline.

The primary outcome will be evaluated using the *x*^2^ test. Confidence interval (CIs) will be calculated using the Clopper-Pearson method. For other Categorical variables, comparisons between treatment groups will assess using the Fisher exact test or Wilcoxon rank-sum test as appropriate. Continuous variables will be performed using the *t*-test or Wilcoxon rank-sum test as appropriate. AE incidences for each treatment group will be compared using Fisher's exact test.

### Quality Control

All staff members will undergo training prior to the trial. The acupuncturists in this trial will have an acupuncture license with at least 2-year acupuncture experience. Monitors will check the case report forms and the acupuncture operation regularly. To improve adherence to intervention protocols, the majority of patients will come from the inpatient setting. The outcomes will be evaluated by independent assessors who are unaware of the group allocation. The data will be input by a clinical research coordinator according to the contents of CRF using the Electronic Data Capture System (EDC), which will be monitored by Clinical Research Associate. Detailed documentation of drop-outs and withdrawals, including the reasons, will be obtained throughout the trial. All of the investigators will always maintain a strict privacy policy to protect confidentiality before, during and after the trial.

### Patient and Public Involvement

The research question was first proposed by two cancer patients suffering OIC. Once the patient has completed the trial, the burden of the intervention will be assessed by patients themselves. All participants will obtain the copy of the completed manuscript if they would like to receive a copy of the trial results.

### Ethics and Dissemination

This study was planned in accordance with the Helsinki Declaration and has been approved by the Ethical Committee of the Guang'anmen Hospital, China Academy of Chinese Medical Sciences (Protocol Approval No. 2018-164-KY-01). The trial was registered at ClinicalTrials.gov (NCT03797586). All eligible patients will be fully informed the duration and procedure of this study, the right to decline to participate during screening period or withdraw from the research at any time. They will have enough time to ask details of the trial and decide whether to participate or not. Any modifications to the protocol that may impact the conduct of the study will be agreed upon by the study investigators and approved by the local ethics committee prior to implementation. Participants will be asked to provide the informed consent form prior to enrollment. The findings of the study will be disseminated by publications in an international peer-reviewed medical journal, with online access. We also plan to present it in relevant national/international conferences.

## Discussion

Constipation is one of the most common and bothersome symptoms among cancer patients receiving opioid for pain management. Some patients would choose to endure the pain rather than the constipation cause by long-term opioid therapy (9). Therefore, it is critical for patients receiving long-term opioids therapy to find a treatment that can improve the gastrointestinal function while not interfere with opioid-analgesia strategy (16). Several previous studies (23, 24) have reported that acupuncture therapy can improve bowel movements in patients with functional constipation. However, clinical trials that have been properly designed to investigate the efficacy and safety of EA for cancer individuals with OIC are lacking. In this trial, a sham control is used to evaluate the efficacy of acupuncture on cancer individuals with OIC, together with the strict design to ensure the reliability of RCT. The results of this study will provide stronger evidence as to whether EA is an effective treatment for OIC among cancer patients.

OIC is characterized by a reduction in bowel movement frequency, straining, and a feeling of incomplete evacuation that is associated with opioid use. In this trial, we will not limit the type of cancer. Our major reason is because it is difficult to recruit when cancer is limited to certain types. Only those patients with good general condition diagnosed with OIC will be included. Oncologists or gastroenterologists are responsible for diagnosing OIC and judging the life expectancy of participant. One of the reasons is that in studies conducted with patients in poor general condition are difficult to maintain the follow up. Considering the severity and irreversibility of OIC, we will select SBMs rather than CSBMs as the primary outcome in this trial. SBMs may also provide an objective reflection of bowel movement improvement for OIC, and has been selected as the primary outcome measurement in several trials (41) among both cancer and non-cancer patients. We will also observe the CSBMs, mean Bristol Stool Form Scale score for stool consistency, mean score for straining, PAC-SYM, PAC-QOL, and patients' global assessment of treatment efficacy as the second outcomes, which may provide more evidences for the efficacy of EA on OIC in patients with cancer pain.

As this trial is targeted at cancer patients who are receiving opioid prescriptions for pain management, it will be a challenge for them to comply well with the frequent attendance to hospital at the treatment and follow-up periods. Hence, the majority of patients will be recruited in the inpatient setting, and the 8-week treatment period and 8-week follow-up period have been designed to be relatively short. The use of rescue medication will further reduce dropout rates. The trial also has several limitations. First, all patients recruited in this study are Chinese, some of whom may have prior experience with acupuncture. The intervention in this trial is EA involving manual acupuncture and electrical stimulation. In order to achieve maintain the blinding of patients, we chose minimal needling at non-acupoints with electrical stimulation for 30 s as the control methods. SA thus may have a few biological effects, which may lead to a false negative result. Second, the acupuncturist will not be blinded to the group allocation due to the nature of the acupuncture. This might result in bias and influence the results. Third, as this study was conducted primarily in the inpatient settings, our results may not be generalized to all OIC populations.

Trial status: Recruitment started in May 2019, and is currently ongoing.

## Ethics Statement

The studies involving human participants were reviewed and approved by Institutional Review Boards of Guang'anmen Hospital in China. The patients/participants provided their written informed consent to participate in this study.

## Author Contributions

ZL and WW conceived the idea of this trial and the design this study. YL is responsible for statistical analysis. This manuscript was drafted by WW and XW and revised by YL, ZL, YS, XL, and YY. All authors have read and approved the final manuscript.

## Funding

This study was supported and funded by the 2019 National Administration of Traditional Chinese Medicine Project of building evidence-based practice capacity for TCM–Project BEBPC-TCM (No. 2019XZZX-ZJ). The funders had no role in the planning, study design, execution, or analysis of the work.

## Conflict of Interest

The authors declare that the research was conducted in the absence of any commercial or financial relationships that could be construed as a potential conflict of interest.

## Publisher's Note

All claims expressed in this article are solely those of the authors and do not necessarily represent those of their affiliated organizations, or those of the publisher, the editors and the reviewers. Any product that may be evaluated in this article, or claim that may be made by its manufacturer, is not guaranteed or endorsed by the publisher.

## Abbreviations

OIC: Opioid-induced constipation
SBMs: spontaneous bowel movements
CSBMs: complete spontaneous bowel movements
PAC-SYM: Patient Assessment of Constipation-Symptom questionnaires
PAC-QOL: Patient Assessment of Constipation-Quality of Life questionnaires.
